# Experimental Detection and Measurement of Crack-Type Damage Features in Composite Thin-Wall Beams Using Modal Analysis

**DOI:** 10.3390/s21238102

**Published:** 2021-12-03

**Authors:** Josué Pacheco-Chérrez, Diego Cárdenas, Oliver Probst

**Affiliations:** 1 Tecnologico de Monterrey, School of Engineering and Sciences, Eugenio Garza Sada 2051, Monterrey 64849, Mexico; a00824133@itesm.mx; 2 Tecnologico de Monterrey, School of Engineering and Sciences, Av. Gral Ramón Corona 2514, Zapopan 45201, Mexico; diego.cardenas@tec.mx

**Keywords:** damage detection, laser vibrometer, composite structures, modal parameters

## Abstract

An experimental proof-of-concept for damage detection in composite beams using modal analysis has been conducted. The purpose was to demonstrate that damage features can be detected, located, and measured on the surface of a relatively complex thin-wall beam made from composite material. (1) Background: previous work has been limited to the study of simple geometries and materials. (2) Methods: damage detection in the work is based on the accurate measurement of mode shapes and an appropriate design of the detection mesh. Both a method requiring information about the healthy structure and a baseline-free method have been implemented. (3) Results: short crack-type damage features, both longitudinal and transverse, were detected reliably, and the true length of the crack can be estimated from the damage signal. Simultaneous detection of two cracks on the same sample is also possible. (4) This work demonstrates the feasibility of automated damage detection in composite beams using sensor arrays.

## 1. Introduction

Composite structures are widely used in mechanical, aerospace, and civil engineering fields. Damage in structures can occur and accumulate during their lifetime. In beam-like structures, cracks are one of the most common forms of damage [1], while periodic manual inspection of structures is still by far the predominant approach to damage detection in practice, in many locations and settings (e.g., off-shore wind turbines), manual inspection may be challenging and costly. To ensure operational safety in such environments, structure structural health monitoring (SHM) techniques based on nondestructive evaluation (NDE) techniques may be a cost-effective alternative, providing the additional benefit of providing continuous feedback to the operator. This paper focuses on vibration-based SHM techniques, where crack-type damage can be detected and measured through changes in dynamical characteristics. Such methods are conceptually simple and robust and tend to be more cost-effective than competing methods. Vibration-based SHM techniques are mainly based on the measurement of modal information such as damping factors, frequencies, and mode shapes [2]. Methods based on changes in mode shapes in particular are suitable for not only accurately detecting but also measuring the length and orientation of crack-type damage features [3].

With the advent of laser vibrometer techniques, modal analysis in general has become easier and more accurate, since these devices operate remotely and avoid the placement of accelerometers on the surface; they are the typical choice in traditional modal analysis. On the downside, laser vibrometers measure the motion along only one coordinate, which may be a limitation in some cases. Laser Doppler vibrometers (LDV) and scanning laser Doppler vibrometers (SLDV) are widely used in the vibration community, because they provide non-contact and spatially dense vibration measurements. In order to determine modal patterns, the surface of interest is divided into a measurement grid, and the laser spot from an SLDV stays at one point long enough to acquire sufficient vibration data and then moves to the next one [4]. With the information of the measurement grid, it is possible to obtain the modal frequencies and mode shapes of the structure. Several methods for damage detection based on modal parameters have been reported in the literature. In different works, natural frequencies, curvature mode shapes, and modal analysis have been applied to damage detection in one-dimensional beams [2,3,5,6,7,8].

Until recently, most of the work on damage detection with modal analysis has focused on damage in simple beams. Lately, some work on damage detection in 2D structures has also emerged, both in experiment and simulation. In a relatively early work, Yang and Oyadiji used modal frequency surfaces for damage detection in simulated glass/epoxy plate-like structures of 550 mm × 550 mm × 4 mm [9]. Their finite element model contained 44 × 44 × 6 elements along the length, width, and thickness directions, and damages of sizes 50 mm × 50 mm and 100 mm × 100 mm were analyzed. In another computational study, Pacheco-Chérrez et al. [10] investigated crack-type damage features on the surface of a composite thin-wall beam (CTWB), showing that the method was capable of locating and measuring the length and orientation of the damage feature, considering high background noise levels. Their technique included a wavelet-based noise-reduction stage and a two-dimensional continuous wavelet transform (CWT), as well as a 2D curve fitting approach for the measurement of the damage features. In the study of Abdulkareem et al. [11] a two-dimensional CWT was applied to decompose the curvature difference between damaged and undamaged first mode shape signals. An experimental investigation was carried out using a square steel plate of 560 mm × 560 mm × 3.2 mm; two accelerometers were used to measure vibrations in the structure, and the size of the damaged analyzed was 80 mm × 80 mm. Govinddasamy et al. [12] used the modal curvature (MC) technique for the detection of large crack-type damage features with sizes of 25 mm, 75 mm, and 125 mm in a plate structure with a length of L = 500 mm and a width of W = 250 mm. The experimental work was performed with an accelerometer and an impact hammer.

In recent works, several groups of authors have used SLDV to obtain the mode shapes and subsequently perform damage detection. W. Xu et al. [13] used a combination of wavelet processing and modal curvature to experimentally detect cracks of 250 mm in an aluminum plate of 1000 mm × 1000 mm. The authors used an SLV Polytec PSV-400 with 443 × 443 measurement points. In another work, the authors used the derivatives of laser-measured mode shapes to detect and locate a notch of 20 mm × 20 mm on an aluminum plate of 400 mm × 600 mm using a mesh of 39 × 39 measurements [1].

In [14], the authors used a 2D wavelet transform to detect damage in composite and aluminum structures. The composite plate analyzed had dimensions of 150 × 300 × 1 mm${}^{3}$, and the scanning area measured with a SLDV was 140 mm × 280 mm at a spatial resolution of 0.5 mm. An SLDV was also used in [15], where the lifting method and the curvature mode shape (CMS) metric were applied to identify structural damage in an aluminum cantilever beam. In another work, Katunin presented an approach for damage identification in beams and plates based on the S-transform [16]. For the experimental part, the author used glass–fiber-reinforced polymeric (GFRP) plates with a length of 300 mm. The vibration measurements were performed with a LDV Polytec PSV-400, and the number of measurement points was 4096 (64 × 64). The authors of [17] used Polytec PSC-400-3D SLDV measurements to detect and locate damage in a metal plate of 150 mm × 400 mm, and the measured grid used was 21 × 11 points. In this work, the location of the damage was only possible in the simulation. In the work of Zhang et al. [18], experimental modal testing was conducted using a scanning laser vibrometer on five CFRP curved plates (one intact and four with damage) of 250 mm × 250 mm × 2.12 mm. A grid of 338 measured points (26 × 13) was used.

A compilation of the most relevant work on damage detection with modal analysis using laser-based vibration measurements is shown in Table 1. Even though there are now a number of works on damage detection in 2D structures, most of them have been conducted under rather idealized conditions. Firstly, most works use simple materials such as aluminum or steel. Secondly, most of the recent work that investigates composite material does so in simple structures such as plates. Thirdly, in most reported work, the size of the damage feature is relatively large. Furthermore, finally, the measurement grids used to probe the structure are often relatively fine-grained. The last point in particular may be limiting factor for many practical applications, particularly if dynamic applications are considered. It also limits the transfer of the methodologies developed to measurement technologies other than scanning laser vibration detection, e.g., sensor arrays embedded in the structure; while some sensors (such as fiber optic devices) allow for quasi-continuous measurements, at least in one direction, sensor arrays based on discrete elements will typically have a relatively coarse mesh size. To address this issue, in the present work, a systematic exploration of the impact of the mesh size on detection accuracy has been conducted, and the final selected mesh was coarse enough for the methodology to be transferable to sensor arrays. This ability to detect crack-type damage feature with a coarse measurement grid is one of the contributions of the present work. The ability to actually measure the length of a crack, both for longitudinal and transverse crack orientations with respect to the beam-like test structure, is another of the contributions of this work. In most other work, one-off damage detection demonstrations have been conducted but systematic assessments are scarce. Furthermore, finally, the test structure considered in the present work is a thin-walled beam structure built from composite material, which can be considered a realistic proxy for practical structures such as wind turbine blades, as opposed to simple beams and plates studied in previous work.

## 2. Methodology Overview

The present work can roughly be divided into three parts: (1) a numerical exploration of the effect of the mesh design and density on the accuracy of the damage signal obtained, (2) a systemic numerical study into the capabilities of the proposed modal analysis methods to detect crack-type damage features of different lengths and orientations on the composite thin-wall beam (CTWB) studied in this work, and (3) an experimental validation of the method proposed using laser vibrometry.

### 2.1. General Metrics

Throughout this work, two metrics previously reported in the literature were used. The first is the mode shape difference (MSD), calculated from the difference of the mode shapes of the damaged and the undamaged structure. Evidently, this method requires prior information of the mode shapes of the undamaged structure. In cases where structural health monitoring (SHM) is considered part of the design, an initial characterization may be possible. In others, a digital twin of the structure may be able to provide the necessary baseline; see, e.g., [3]. In the present work, an initial characterization of the undamaged structure was conducted, and the results were stored for later use. The second metric used is the curvature of mode shapes (CMS); this method only requires the mode shapes of the damaged structure.

#### 2.1.1. Mode Shape Difference

The mode shape difference (MSD) is obtained by subtracting the mode shape of the undamaged structure $\varphi_{r}^{\left(u\right)}$ from the undamaged structures’ mode shape $\varphi_{r}^{\left(d\right)}$ for a given mode *r* [11] and averaging over all modes in the range considered [3].

(1) $$\mathrm{MSD}\left(z,p\right)=\sum_{r=1}^{N}\frac{\varphi_{r}^{\left(d\right)}\left(z,p\right)-\varphi_{r}^{\left(u\right)}\left(z,p\right)}{N},$$

where *N* is the number of mode shapes, and $\varphi_{r}^{\left(u\right)}\left(z,p\right)$ and $\varphi_{r}^{\left(d\right)}\left(z,p\right)$ are the values of the nodal displacement at nodes (z,p) in the rth mode of the undamaged (*u*) and damaged (*d*) structure, respectively. The two coordinates in the present case refer to the longitudinal or axial direction *z* and the circumferential direction *p* of the thin-wall beam structure studied (Figure 2) and are described below.

#### 2.1.2. Curvature of Mode Shapes

An alternative to the MSD metric is the determination of the curvature of mode shapes. The curvature is calculated along the *z*-axis (see Figure 2). The curvature of mode shape (CMS) is calculated by a central difference approximation [20]:

(2) $$\phi_{z}^{\prime \prime }=\frac{\left(\phi_{z-1}+\phi_{z+1}-2\phi_{z}\right)}{h^{2}},$$

where $\phi_{z}$ is the mode shape at the mesh point *z*, and *h* is the distance between neighboring mesh points. In this work, the average of the CMS values of all modes *r* is used:

(3) $$\Phi =\frac{1}{N}\sum_{r=1}^{N}\phi_{z}^{\prime \prime ,r}$$

In the case of both metrics, the result of the assessment is a damage map D(z,p) of the two spatial coordinates *z* and *p*, where the damage feature is expected to stand out as a peak or bump on an otherwise flat background.

### 2.2. Metrics Used to Assess the Effect of Different Mesh Sizes

The number of vibration measurement points directly impacts the time required for data acquisition, processing, and storage. In the case of sensor arrays embedded in the structure, they also imposed practical limits on the design of the measurement system. Therefore, the design of a mesh with a reduced number of points that still allows to retain the necessary amount of information for the detection and measurement of damage is an important practical concern.

The idea proposed in this work is to assess the *similarity* between an ideal damage map $D_{0}\left(z,p\right)$ and a practical damage map D(z,p) sampled at a reduced resolution and make a decision on the optimal practical mesh design based on the inspection of the similarity metric. Two metrics were used in the present work: (1) the Wasserstein-2 distance (WD), also referred to as Frechet Inception Distance (FID), a metric proposed by [21], and (2) the mean square error (MSE).

### 2.3. Fréchet Inception Distance or Wasserstein Metric

M. Fréchet introduced the metric now named after them in the context of probability distributions with given first and second moments [22]. The Fréchet distance d(F,G) between two distributions *F* and *G* in 1D is defined by:

(4) $$d^{2}\left(F,G\right)={\left(\mu_{x}-\mu_{y}\right)}^{2}+{\left(\sigma_{x}-\sigma_{y}\right)}^{2},$$

where $\mu_{x},\;\mu_{y}$, and $\sigma_{x},\;\sigma_{y}$ are the means and standard deviations of *F* and *G*, respectively. Given that the original Fréchet distance is limited to one-dimensional functions, a generalization to two dimensions is required in the present case, where the distance between two images (the damage maps $D_{0}\left(z,p\right)$ and D(z,p)) is required. Such a generalization is given by the squared Wasserstein metric, also know as the Fréchet Inception Distance (FID). In general, the FID is defined on the space of probability distribution on $R^{n}$ having second moments [23]. When *F* and *G* belong to a family of *n*-dimensional distributions, the FID is given by

(5) $$d_{\mathrm{FID}}^{2}\left(F,G\right)={\left|m_{x}-m_{y}\right|}^{2}+\mathrm{tr}\left(C_{x}+C_{y}-2{\left(C_{x}C_{y}\right)}^{\frac{1}{2}}\right),$$

where $m_{x},\;m_{y}$ and $C_{x},\;C_{y}$ are the means and covariance matrices of *F* and *G*, respectively.

### 2.4. Mean Square Error

The mean square error (MSE) measures the average of the squares of the errors, i.e., the average squared difference between the estimated values and the actual value.

(6) $$d_{\mathrm{MSE}}^{2}=\frac{1}{np}\sum_{i=1}^{np}{\left(F-G\right)}^{2},$$

where np is the number of data points in the image (np = number of rows × number of columns = $n_{r}\times n_{c}$), and *F* and *G* are the images to be compared.

### 2.5. Data Acquisition and Processing

**Figure 1:**
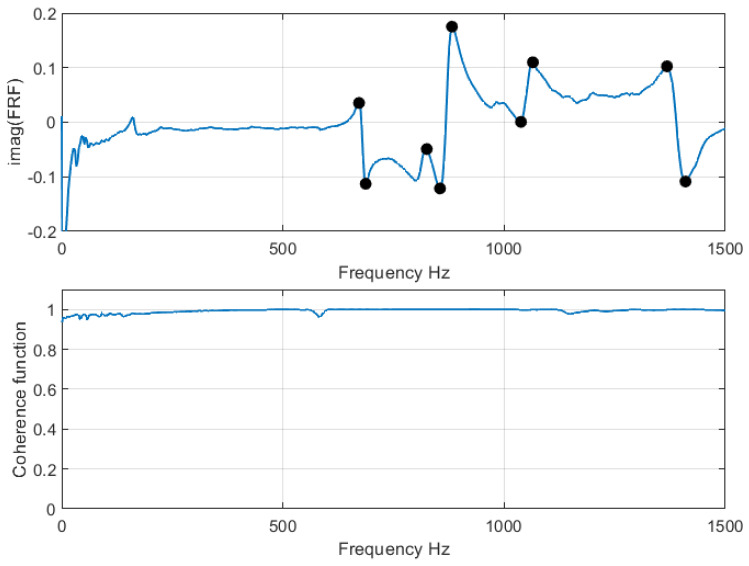
Frequency response function and coherence function for the center point at the top center of the structure.

For each signal in the different measurement points, the frequency response function (FRF) was obtained. The FRF is calculated from the relation between the excitation signal *x* and the response signal *y* in the frequency domain. In this study, the cross-power method is used to obtain the FRF:

(7) $$H_{xy}=\frac{G_{xy}}{G_{xx}},$$

where $G_{xy}$ is the averaged cross power spectrum between the input signal *x* and the response *y*, and $G_{xx}$ is the averaged auto-spectrum of excitation signals. To evaluate the quality of the FRF measurements, the coherence function defined by the following equation was used:

(8) $$C_{xy}^{2}=\frac{{\left|G_{xy}\right|}^{2}}{G_{xx}G_{yy}},$$

where $G_{yy}$ is the averaged auto-spectrum of the response. For the coherence function, more than one average is necessary. The coherence function has values less than or equal to 1, with a value of one indicating an ideal measurement process. In this study, an average of ten measurements were calculated at each measurement point. Figure 1 shows the imaginary part Im(Hxy) of the FRF as well as the coherence function for a point at the central upper part of the ellipse. The peaks in Im(Hxy), shown as black circles in the figure, indicate the natural frequencies of the structure. These frequencies were obtained for each measurement point in the structure, and the experimental mode shapes were calculated using the quadrature picking method [24]. It can be seen in the figure that the value of the coherence function is close to one at all frequencies, indicating that individual measurements are very consistent with each other. It should be pointed out that obtaining a consistently high coherence function is not trivial, particularly if a manual excitation (typically with an impact hammer) is used, since a very steady hand of the experimenter is required. A common alternative is the use of a shaker, providing the added benefit of an excitation with a programmable frequency spectrum. However, the use of a shaker requires a fixed coupling between the shaker and the structure under study, which provides a path for background noise and may also restrict the response of the structure. To improve upon the problems described, an automated impulse excitation was implemented in the present work, where the excitation tip, equipped with a force sensor, was programmed to periodically hit the structure under study by means of an actuator.

### 2.6. Sample Geometry and Simulation Setup

**Figure 2:**
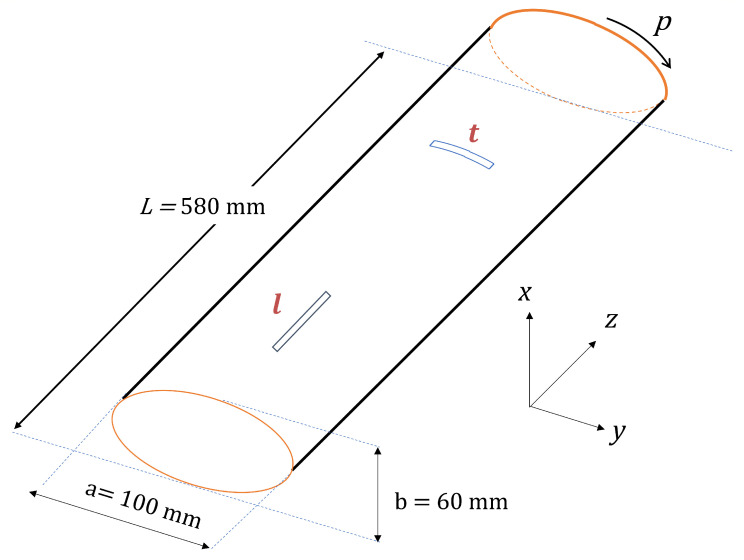
Geometry of the structure and nomenclature of the damage features studied.

A thin-walled hollow elliptical cylinder was used as the sample both in the numerical simulations as well as in the experimental validation; its geometry is shown in Figure 2. The cylinder dimensions are: a major axis of 100 mm, a minor axis of 60 mm, and a length (in the *z* or longitudinal direction) of 585 mm. The walls of the cylinder have composite layered configurations of ${\left[10\right]}_{2s}$ and ${\left[45\right]}_{2s}$ of unidirectional laminate (UDL), with each layer having a thickness of 2 mm. The material properties employed in the FE analysis are given in Table 2; these properties are consistent with those used in the physical prototypes.

The numerical simulation was set up and conducted in the commercial finite-element (FE) modeling software suite, Mechanical APDL 2020. The modal analysis tool of the APDL suite was used to extract the natural frequencies and mode shapes of the structure.

The surface of the beam shell can be conveniently represented in θ-*z* plane, where (r,θ,z) are cylindrical coordinates, or alternatively, in the *p*-*z* plane, where *p* is the arc-length coordinate at the outer edge of the shell:

(9) $$p\left(\theta \right)=\sqrt{1+{\left(\frac{dy}{dx}\right)}^{2}}\;dx=\frac{1}{a}\int_{\theta_{0}}^{\theta }\sqrt{a^{4}-a^{2}\left(a^{2}-b^{2}\right)\sin^{2}\theta }\;d\theta ,$$

where x=asinθ, dx=acosθdθ, a=100 mm, and b=60 mm. The *p*-*z* representation was used for both visualization and crack length assessment.

## 3. Determining the Optimal Mesh Size

In order to design a measurement mesh that would allow for the detection of the smallest crack-type damage features considered in this work while reducing the number of measurement points as much as possible, a systematic assessment of the effect of the mesh was conducted. As mentioned above, the search for an efficient measurement mesh was motivated both by practical concerns for the present work, such as a reduction in the required data acquisition time and the applicability of the concept to other measurement settings, such as embedded sensor arrays.

The design process was based on numerical studies of modal response of the structure for different mesh sizes, using the setup described in Section 2.6. To assess the effect of the measurement mesh, virtual measurement points were selected by re-sampling the modal surfaces, obtained on the slightly irregular grid of the FE model, at a regular grid with reduced resolution. For a given number of measurement points $n_{r}$ in the circumferential direction (*p*), the number of points $n_{c}$ in the longitudinal or axial direction (*z*) was varied, and vice versa. For each pair $\left(n_{r},n_{c}\right)$, both the FID metric $d_{FID}^{2}$ and the mean square error $d_{MSE}^{2}$ between the damage map $D_{0}\left(z,p\right)$ calculated at the native resolution of the FEM simulation and the damage map $D(z,p;n_{r},n_{c})$ obtained at a coarser resolution were calculated. For ease of viewing and comparison, the damage maps obtained with reduced meshes were interpolated to the same number of points as the full mesh.

**Figure 3:**
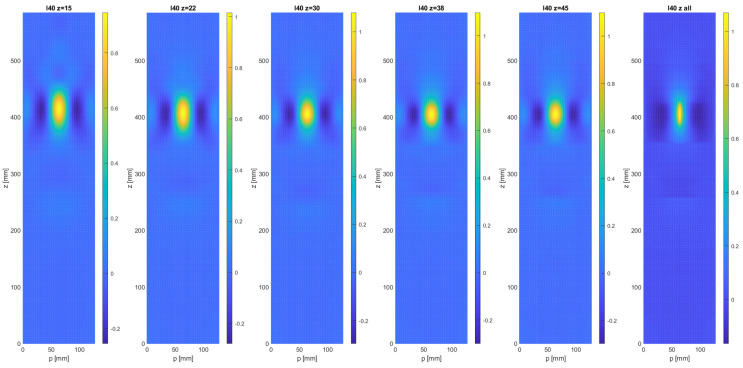
Evolution of the damage map D(z,p) of the upper surface of the composite thin-walled test beam, generated with the MSD metric, for varying numbers of sample points in the axial beam direction (*z*).

In Figure 3, the effect of reducing the mesh resolution in the *z*-direction is shown for the case of the native resolution in the circumferential direction (*p*). The number of grid points chosen in the *z*-direction were $n_{c}=$ 15, 22, 30, 38, and 45. The number of points in the *p*-direction was $n_{r}$ = 5 in all cases, with the exception of the image with the native resolution (far right). These numbers have to be compared to the native resolution of the FE model, based on 13,272 nodes, with an approximate number of points in the *z*-direction of $n_{c,0}=$ 237 and a number of $n_{r,0}=$ 56 points in the *p*-direction.

**Figure 4:**
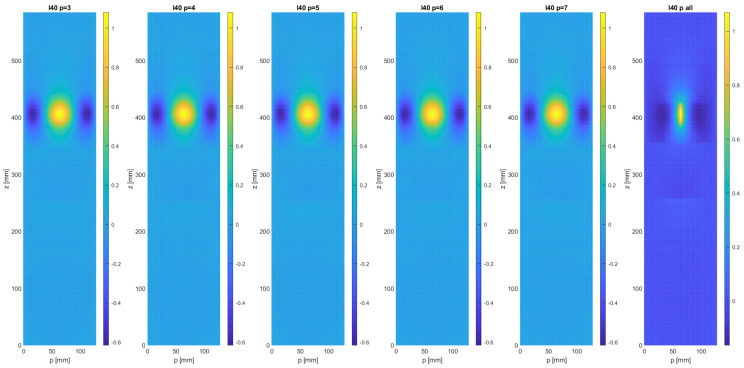
Evolution of the damage map D(z,p) of the upper surface of the composite thin-walled test beam, generated with the MSD metric, for varying numbers of sample points in the circumferential direction (*p*).

It can be seen that the main effect of the re-sampling process appears to an increase in the crack width, and a modest increase in the apparent length of the crack until $n_{c}$ = 30, as indicated by the highest values of the MSD signal. For an even coarser sampling resolution, the increase in apparent crack length becomes more notorious. The effect of a variation in sampling density in the circumferential direction can be seen in Figure 4, where down-sampled MSD damage maps are shown for $n_{c}=$ 30 and $n_{r}=$ 3, 4, 5, 6, and 7. As expected, the damage signal from the crack is spread out perpendicularly to the crack, but the effect of reducing the sampling density below 7 measurement points is relatively modest.

**Figure 5:**
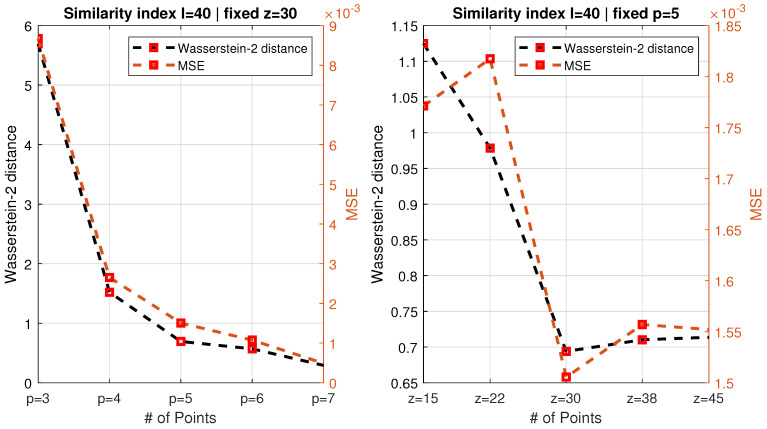
Evolution of the similarity metrics (FID or Wasserstein-2 distance, as well as MSE) as function of the number of measurement points in the axial (*z*) direction for a fixed number of circumferential measurement locations (**left**) and for a variable number of points in the *p*-direction, for a fixed number in *z* (**right**). In all cases, the crack was longitudinal (pointing in the *z*-direction), with a length of 40 mm.

In order to reach conclusions regarding the optimal sampling mesh, the similarity metrics introduced above were evaluated for all cases. For both FID and MSE, lower is better, and a value of zero corresponds to perfect similarity. The results for a longitudinal crack with a length of 40 mm are shown in Figure 5. The left graph shows the effect of increasing the number of circumferential sampling points (from three up to seven). It can be seen that the Wasserstein-2 and the MSE metric yield very similar results in this case. The biggest improvement in similarity is obtained by increasing $n_{r}$ from three to four, followed by a still substantial improvement from four to five. A further increase in sampling density only yielded marginal improvements. Similarly, in the case of the longitudinal resolution, an increase from $n_{r}$ = 15 to 22 and, further, to 30 improves the similarity between the damage map in its native resolution and the down-sampled map very substantially, with a further increase to 38 and 45 not providing any extra improvement. Note that the Wasserstein-2 metric appears to provide a more consistent measure of similarity that the mean square error.

## 4. Numerical Study

A numerical study based on finite-element simulations was conducted first. The same composite thin-wall structure, a hollow elliptical cylinder, was considered for both the numerical and the experimental study. In both cases, a free-free configuration was used, i.e., both ends were allowed to move freely, while in the numerical simulations a cantilever configuration could be simulated easily, in the laboratory setup it is more difficult to isolate vibrations from the building, which is why it was decided to suspend the beam elastically to allow for cleaner measurements.

**Figure 6:**
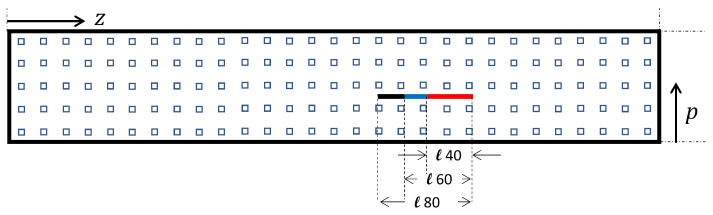
Damage locations and measuring points.

Based on the findings of Section 3, a 5 × 30 grid was used in the *p*-*z* plane (see Figure 6). At each point of the re-sampled mesh, the modal displacement along the *x* coordinate (see Figure 2) was extracted from the numerical model. The fault analyzed is located between the third and fourth point of the *p*-axis in the upper part of the structure and has a width of 2 mm, a depth of 4 mm, and comes with three different lengths, *ℓ*40 = 40 mm, *ℓ*60 = 60 mm, and *ℓ*80 = 80 mm. The fault locations in the *p*-*z* plane are given in Table 3.

### 4.1. Numerical Results

**Figure 7:**
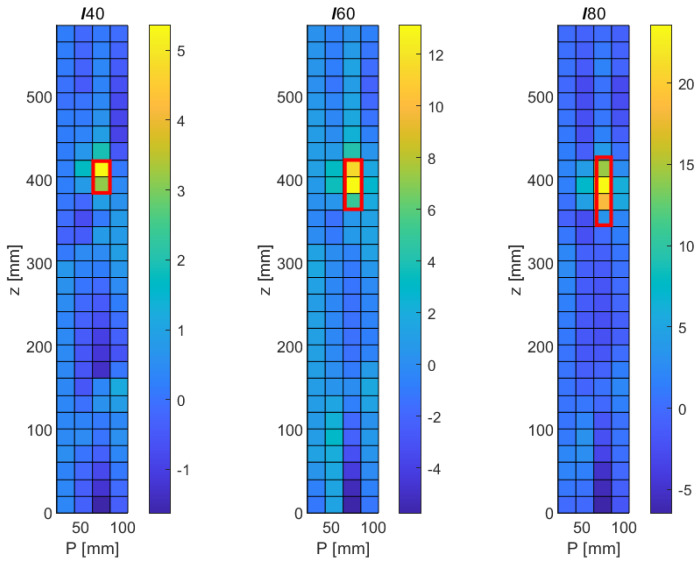
Numerical results obtained with the MSD method for longitudinal cracks of varying lengths (*ℓ*40, *ℓ*60, and *ℓ*80). **Left**: 40 mm crack, **middle**: 60 mm crack, **right**: 80 mm crack.

Figure 7 shows the numerical results for the different longitudinal faults (*ℓ*40, *ℓ*60, and *ℓ*80) obtained with the MSD method in the *p*-*z* plane. Regions with the highest differences in modal displacements between the original and the damaged structure are shown in yellowish colors; regions with little changes appear in bluish colors. The red frames superimposed on the subfigures indicate the expected size and location of each of the faults. Referring to Table 3, it can be verified that the locations of the fault fall into the ranges of the down-sampled cells in the figure. The circumferential position of all three longitudinal faults is at p= 73 mm, which falls into the range of the third circumferential class between 50 mm and 75 mm shown in the graph. Similarly, the crack can be seen to extend down from z= 420 mm in all three cases, extending to 380 mm, 360 mm, and 340 mm. It can be verified that the modal displacement difference in these ranges is particularly pronounced in each of the cases. It can therefore be concluded that the locations of the three faults *ℓ*40, *ℓ*60, and *ℓ*80 are correctly detected, at least under the idealized conditions of a numerical study with vanishing noise level. It should be noted that some changes in the modal displacement also occur far away from the fault, e.g., towards the lower end of the structure. However, these changes are relatively small and show the opposite sign of the change occurring at the fault. Given that modal responses are a collective phenomenon by definition, a certain global effect of a local disturbance is, of course, to be expected. However, it is clear from the the figure that this effect is minor, and that the modification of the modal pattern is mostly confined to the area of the fault.

**Figure 8:**
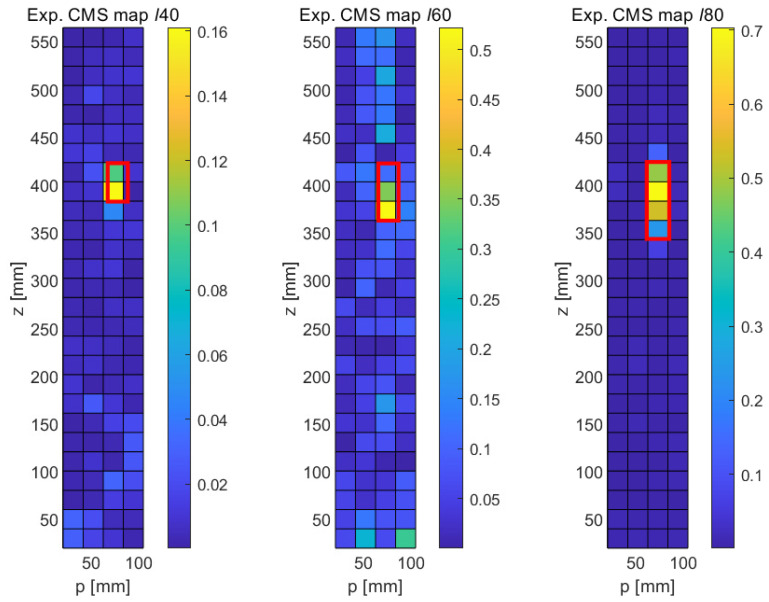
Numerical results obtained with the CMS method for longitudinal cracks of varying lengths (*ℓ*40, *ℓ*60, and *ℓ*80). **Left**: 40 mm crack, **middle**: 60 mm crack, **right**: 80 mm crack.

As explained in Section 2.1, the MSD requires the knowledge of the mode shapes of the undamaged structure, which may not always be available. The CMS method provides an alternative for those cases. Figure 8 shows the results obtained with this method in the numerical case; as before, the results shown are for the upper part of the elliptical cylinder and are displayed in the *p*-*z* plane. It can be seen that the crack-type damage features show up as bright regions in the CMS maps in a similar way to their appearance in the MSD maps, albeit at a higher noise level. In the case of the shortest crack (*ℓ*40), the fault clearly stands out in its CMS signal, but a relatively large distributed disturbance also occurs in the lower part of the structure. In the cases of the longer cracks (*ℓ*60 and *ℓ*80), significant CMS signals are limited to the damage areas, as expected, but the signal is not quite as clean as in the case of the MSD method. This somewhat poorer performance of the CMS method was, of course, expected, given its lack of a baseline case; however, the fact that the faults are still clearly detectable in the CMS maps is quite encouraging.

**Figure 9:**
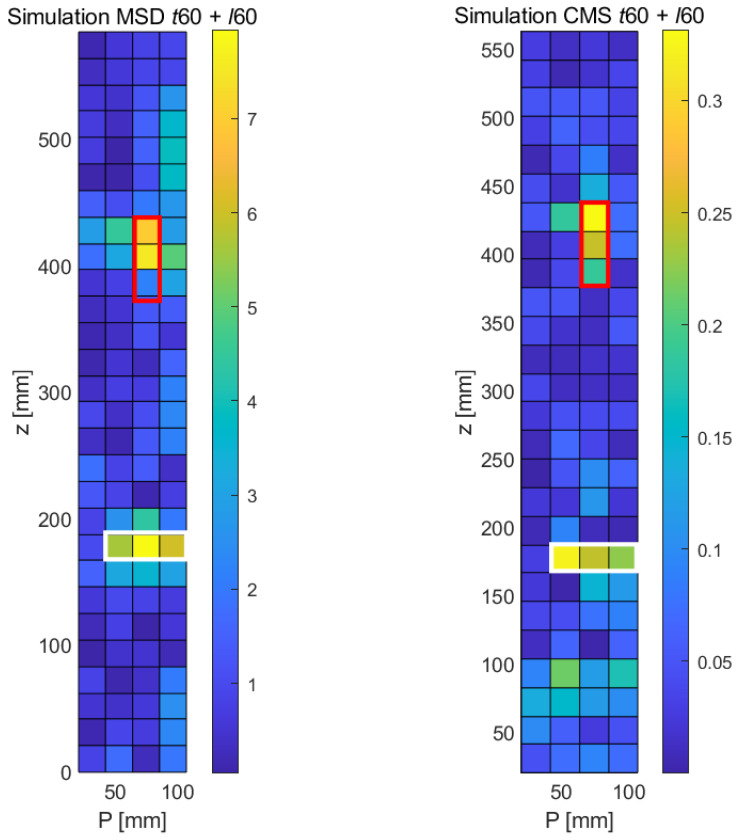
Numerical results obtained with the MSD and the CMS method, respectively, for two simultaneous failures at the sample (ℓ60 and *t*60).

Another important question relates to the capability of the methods to simultaneously detect two or possibly more faults. Though often, damage initiates at locations of maximal stress/strength ratios and propagates from there, several faults may initiate at different locations, e.g., under extreme load conditions. To explore this subject, the simultaneous occurrence of a longitudinal crack of 60 mm (*ℓ*60) located at the same position as before and a transverse crack, also with a length of 60 mm (*t*60) and located at the lower part of the structure, was simulated and later studied experimentally (see Section 5); see Table 3 for the information on the crack locations. Given the collective nature of modal oscillations, as seen before, some modifications of the mode shapes occur far away from the fault do occur, making the simultaneous detection of several faults a non-trivial task. The results of the numerical study, obtained with the MSD and the CSM methods, are shown in Figure 9. The boxes in red and white indicate the size and expected location of the longitudinal and transverse faults, respectively. Interestingly, the overall signature of this double fault is similar in both maps. Both the longitudinal and the transverse crack show up as regions of high (MSD and CMS) signal, though in both methods, the signal of the lower (transverse) crack is somewhat lower. The length of both longitudinal cracks is likely to be underpredicted by most image processing techniques, although a discussion of post-processing aspects is not part of the intended scope of this paper. (For a detailed proposal for the assessment of the length and orientation of cracks as detected by modal techniques, the reader is referred to [10]). The length of the *t*60 is detected correctly by both methods, although the adjacent region of a relatively high CMS signal at the lower part indicates a somewhat poorer performance of the CMS method.

## 5. Laboratory Experiments

### 5.1. Experimental Setup

**Figure 10:**
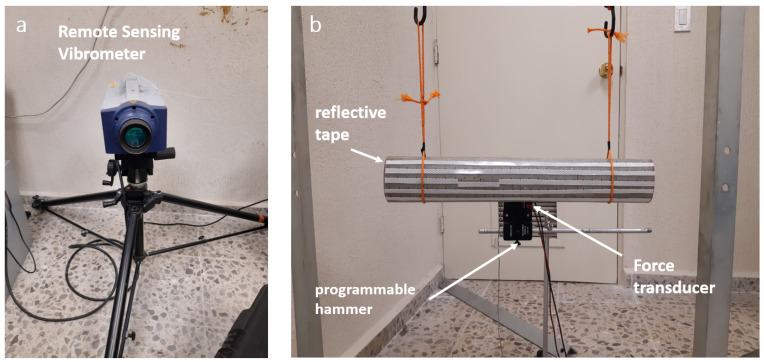
(**a**) Remote sensing vibrometer. (**b**) Experimental Setup using in this work.

The experiments were performed on a composite elliptical cylinder with the dimensions and material properties given in Section 4.1. The cylinder was excited in the middle of the lower face employing a programmable actuated metallic pin connected to a PA253 power amplifier. A Brüel & Kjær 8230 force transducer was attached to the end of the exciter. Vibration measurements were taken at a distance of 5 meters with the RSV-150 Remote Sensing Vibrometer from Polytec (Figure 10a). For the signal acquisition, a dynamic signal analyzer PHOTON + and the RT Pro Photon software were used; the sampling frequency was 4000 Hz. The annotated photographs in Figure 10 show the setup described. By design, laser vibrometers detect the the modal displacement along the laser beam direction. To a good approximation, this direction corresponds to the *x*-coordinate in Figure 2 at all measurement locations, given the large distance between the sample and the vibrometer, and the correspondingly small angle subtended by the horizontally suspended beam (≈0.1 m/5 m $\approx 0.1^{\circ }$). Measurements were taken individually at the 30 × 5 measurement locations determined as optimal in Section 3 by slightly adjusting the angle of the vibrometer. Reflective tape was placed at all locations to ensure a strong signal at the vibrometer. The quadrature picking method [24] was used to construct the mode shapes.

### 5.2. Sample Preparation

**Figure 11:**
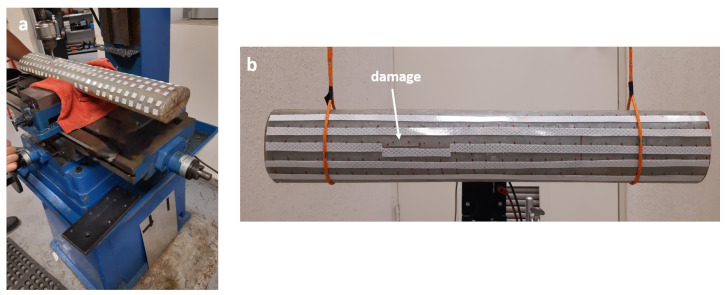
(**a**) Damage milling. (**b**) Longitudinal damage l40.

The damages analyzed in the experiment have the characteristics and locations presented in Table 2 and Table 3. These damages were carried out sequentially in the same structure, starting with the 40 mm one (Figure 11b). After taking the corresponding measurements, the length of the damage was increased to 60 mm and, finally, to 80 mm. The damage feature was created by milling the structure (Figure 11a).

### 5.3. Experimental Results

**Figure 12:**
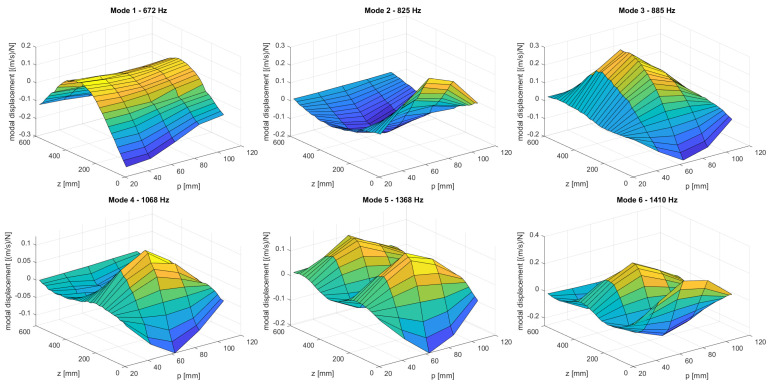
The first six mode shapes obtained experimentally with the undamaged structure.

The experimental mode shapes obtained with the setup described are shown in Figure 12 for the case of the undamaged structure; the mode shapes for the damaged structure appear indistinguishable from the reference modes to the naked eye and are therefore omitted for brevity. Beam-like bending modes can be seen to dominate at the lower modes, and the free movement at both ends is conspicuous. The more complex spatial patterns at the higher modes are also clearly appreciated.

As shown in Table 4, the two lowest natural frequencies respond little to the damage, which is consistent with the large-scale nature of the modal patterns and the small size of the damage features. However, some of the higher modes show a consistent trend towards declining natural frequencies as the size of the damage increases, consistent with a softening of the elastic response as damage progresses.

**Figure 13:**
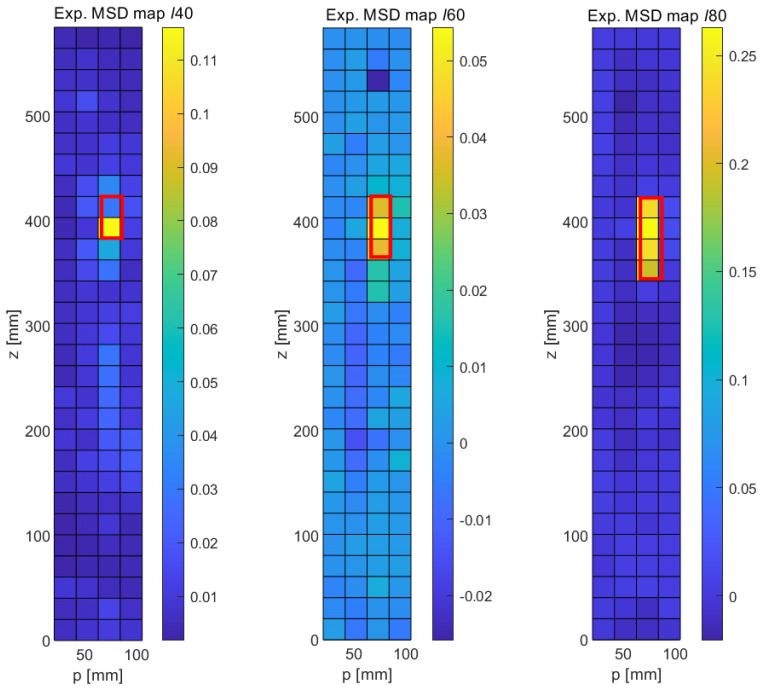
Experimental results obtained with the detection of longitudinal cracks with the MSD method. **Left**: 40 mm crack, **Middle**: 60 mm crack, **Right**: 80 mm crack.

Figure 13 shows the experimental results obtained with the MSD method for the three crack-type damage features ℓ40,ℓ60, and ℓ80 sizes. These results are surprisingly clean results and rival the quality of the numerical results. We first note that the general noise level is very low and fairly uniform. As opposed to the numerical results, no long-range effects are observed, i.e., the changes in modal response are very localized. Furthermore, the MSD metric is practically positive throughout (with the exception of one outlier in the upper part of the central graph of Figure 13). As before, the nominal locations of the cracks have been indicated with red rectangular frames superimposed on the damage maps. It can be seen that a clear signal emerges at the expected locations in all three cases, and the length of the crack is correctly predicted, with the exception of the shortest fault ℓ40, where the implied length is only half of the true length. It can therefore be stated that the MSD correctly detects all three cracks and reproduces the correct crack length in the case of the two longer cracks (ℓ60 and ℓ80).

**Figure 14:**
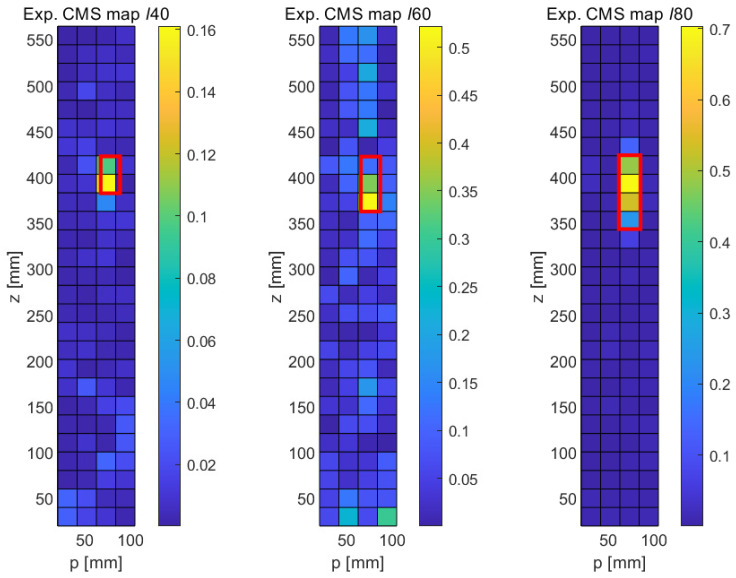
Experimental results obtained with the detection of longitudinal cracks with the CMS method. **Left**: 40 mm crack, **Middle**: 60 mm crack, **Right**: 80 mm crack.

The experimental results obtained with the CMS method are shown in Figure 14. Unsurprisingly, given the lack of a baseline, the CMS results are somewhat noisier in general. However, and this is quite remarkable in an experimental setup, all three faults can be clearly identified. In the case of the longest crack (ℓ80, right damage map in Figure 14), the signal-to-noise ratio is very good, and the fault is clearly distinguished, though its length is slightly underestimated. Similarly, the lengths of the ℓ40 and the ℓ60 are slightly underpredicted. It should be noted that these experimental findings are largely consistent with the numerical results (Figure 8), which also show a higher general noise level and a slight underprediction of the true crack length. In spite of the experimental challenge of individually acquiring FRFs at each of the 5 × 30 locations for each of the three samples, the experimental uncertainties and background noise level in the laboratory appear to have a negligible influence, demonstrating the great potential of carefully conducted laser-based vibrometry measurements.

**Figure 15:**
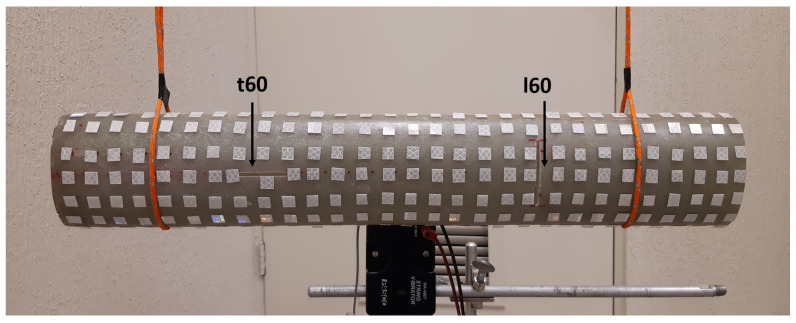
Second experimental sample studied in the present work, prepared with two simultaneous damage features (*t*60 + *ℓ*60).

The remaining step in the laboratory work was the experimental demonstration that two cracks (chosen to be one longitudinal (ℓ60) and one transverse (t60) crack) can be detected simultaneously by either method (MSD or CMS), as previously demonstrated in the FE simulations. Given that the first sample had already been modified with a 80 mm-long crack, in order to assess the response of the damage maps to an increasing crack length, a second sample was prepared for that purpose. This additional hollow cylinder with an elliptical base belongs to the same batch as the previous sample and has nominally identical geometrical and materials properties. A photograph of this second sample, already in its experimental setup, is shown in Figure 15.

**Figure 16:**
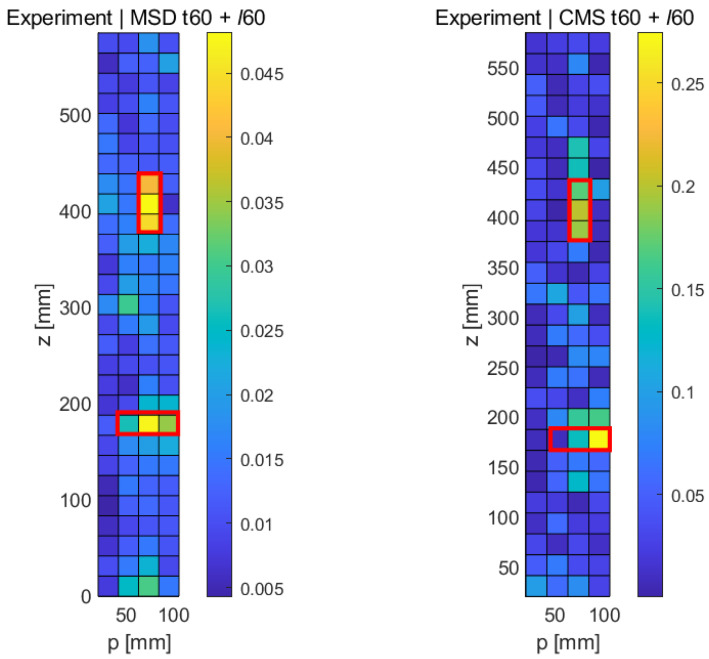
Experimental results obtained with both methods (MSD & CMS) for the case of two simultaneous damage features (*t*60 + *ℓ*60).

The experimental results for the two-fault sample using the MSD method are shown in the left part of Figure 16, with the expected location of the longitudinal fault being shown in the red box and the transverse fault in the white box. It can be seen that the longitudinal crack (*ℓ*60 stands out clearly (yellow/orange colors), whereas the transverse crack in the lower part of the damage map shows a somewhat lower response, while still producing a recognizable damage signal of approximately the correct length. As before, the CMS damage map has a somewhat higher general noise level, but both faults can be detected reliably. The longitudinal crack produces a somewhat lower signal compared to the MSD case, but the crack length can be estimated correctly. The lower (transverse) crack is also detected, but its length is underestimated.

## 6. Discussion

The general research question laid out at the beginning of this work was if it was possible to detect small crack-type damage features in a realistic composite thin-wall beam (CTWB) structure with a relatively coarse measurement grid using modal analysis. The interest in CTWB was motivated by the large range of applications of composite materials and particularly hollow (“thin-wall”) structures in engineering. Though a considerable body of work on fault detection within simple (unstructured) beams exists, and some work has been published in recent years on simple two-dimensional structures (plates), to the best knowledge of the authors, no work has been published so far on three-dimensional structures. The reasons why the extrapolation to more complex bodies such as CWTBs is not trivial are multiple, including a more complex modal structure and larger uncertainties and inhomogeneities in material properties, among others. In the specific case of laser doppler vibrometer (LDV) measurements, practical issues also arise, such as the variation of the local normal and, therefore, variations in the angle of the reflected beam.

**Figure 17:**
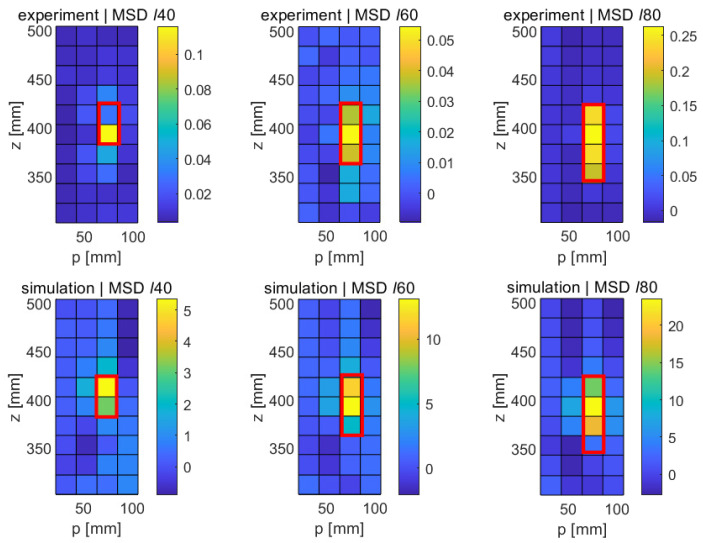
Comparison of the experimental and the numerical results for the case of the MSD method and the three longitudinal cracks of varying length (40 mm, 60 mm, and 80 mm). For clarity, only the affected upper portion of the structure has been shown.

**Figure 18:**
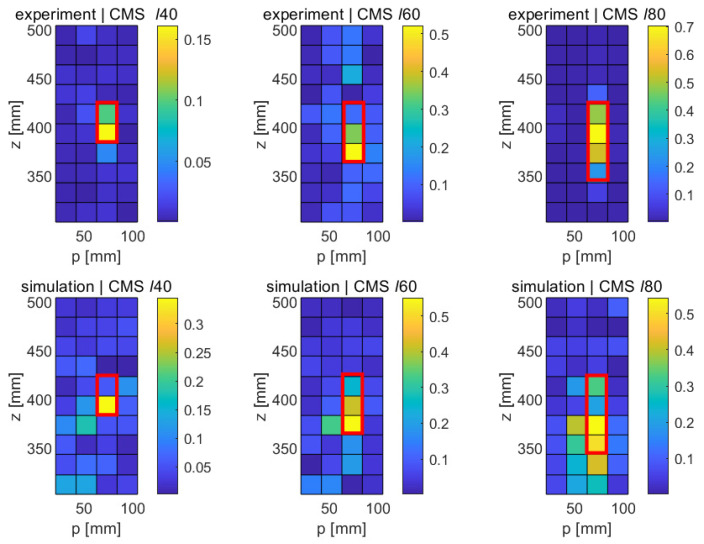
Comparison of the experimental and the numerical results for the case of the CSM method and the three longitudinal cracks of varying length (40 mm, 60 mm, and 80 mm). For clarity, only the affected upper portion of the structure has been shown.

In spite of the challenges mentioned, a successful proof-of-concept has been established, showing not only that relatively small damage features can be reliably detected with the method proposed, but also that the quality of the experimental results is practically indistinguishable from the numerical results obtained with a finite-element model of the thin-wall structure, at least in a laboratory setting where the test structure was carefully suspended to avoid the transmission of noise from the building. The great degree of similarity between the numerical and the experimental results is demonstrated by the juxtapositions in Figure 17 for the case of the MSD method and in Figure 18 for the CMS method.

It can be seen that, in all cases, the results of the FE simulation accurately predict the experimental outcome, and that, in all cases, the fault is correctly detected, down to cracks of 40 mm. The fault length can be estimated correctly for cracks of 60 mm or longer, whereas the length of the 40 mm crack is generally somewhat underestimated. Not unexpectedly, the MSD method, based on the difference between baseline results and the mode shapes of the damaged structure, performs somewhat better than the CMS method, which works on modal data from the damaged structure alone. However, the difference is not dramatic, which is encouraging for practical purposes, since (a) often, the baseline information may not be available, and (b) the acquisition of the baseline case translates into additional measurement time, data acquisition, storage, and processing requirements.

**Figure 19:**
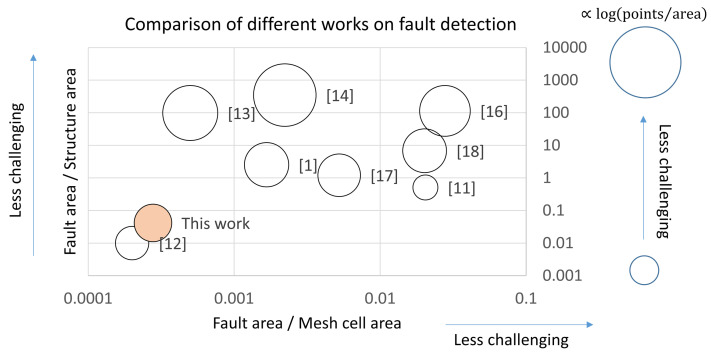
Benchmarking of the present work relative to published work in the literature. (see also Table 1). Horizontal axis: $\alpha =A_{F}/A_{S}$. Vertical axis: $\beta =A_{F}/A_{M}$. Bubble size: ∝log(γ)+const.

Though the present work is, to the best knowledge of the authors, the first demonstration of damage detection in CTWB structures, which singles out the present research from other recent work such as the one reported in Table 1, which is mostly on plates and simple materials, it is still useful to conduct some basic benchmarking to place the contributions of the present work in an appropriate context. To do so, we have calculated three basic metrics for each work on two-dimensional structures (mainly plates, with the exception of the present work, where the modal displacement on the curve upper shell of a CWTB structure has been analyzed): $\alpha =A_{F}/A_{S}$, where $A_{F}$ is the fault area and $A_{S}$ is the full surface area of the test structure; $\beta =A_{F}/A_{M}$, where $A_{M}$ is the area of the unit cell of the measurement mesh; and $\gamma =N/A_{S}$, where *N* is the number of points contained in the measurement mesh. The larger α and β are, the less challenging it is to detect a fault. Similarly, the larger γ is, the easier it should be to detect changes in the modal patterns (see Figure 19).

It can be seen that the present work compares very favorably to the bulk of the previous work, independently of the fact that it deals with a CTWB structure as opposed to simple plates, since it detects a relatively small fault (small α) and does so using a relatively coarse mesh (small β and small γ).

While Figure 19 provides a graphical overview of the nominal performance of each of the methods reported in the literature, it says nothing about the actual accuracy of each reported work. Table 5 provides this information. As seen in the comment column of the table, most authors do not provide actual numerical size estimates of the damage features detected, so size figures were estimated by us based on the evidence presented in each article. It can be seen that all published works, as expected, do detect damage. In most work, the authors were also able to approximately locate the damage, and in several occasions, a good estimate of the size of the damage feature can be obtained from the published material. In others, the discrepancies are either large, or no reliable size measurement was possible. The present work goes beyond the reported state of the art in the sense that not only does it demonstrate the capability of detecting and accurately quantifying damage, but also does so on the curved surface of a thin-wall beam structure, as opposed to the simple plates and beams studied in the literature so far.

## 7. Summary and Conclusions

An experimental demonstration of the feasibility of the detection, localization, and length measurement of crack-type damage features on the curved surface of a thin-wall beam structure made from composite material using modal analysis has been conducted. To the best knowledge of the authors, this is the first demonstration of its kind, since previous work has been limited to simple plates and beams, as well as to qualitative of descriptive analysis of the faults studied. The present work also shows that reliable detection, localization, and measurement of cracks has been achieved a with a baseline-free modal analysis technique, i.e., one where the information of the undamaged structure is not available; however, a method using this baseline information produces slightly more accurate results. It is also shown that experimental modal analysis can be conducted with a level of accuracy that is practically indistinguishable from the results of numerical modeling obtained with a finite-element (FE) model of the structure. This is not only an encouraging endorsement for experimental modal analysis itself, but also paves the way towards using numerical models as a proxy for the baseline information of the undamaged structure, i.e., as a digital twin, in those cases where experimental baseline information is not available. A final note concerns the role of the measurement mesh: a systematic assessment of the mesh size was conducted based on the numerical (FE) model of the structure by downsampling the numerical mode shape maps prior to conducting the experiments, based on similarity metrics between the full (high-resolution) and downsampled mode shape maps. Both the numerical and the experimental studies then confirmed that small crack-type features can be readily detected with a relatively coarse map.

While the present work was conducted with a laser Doppler vibrometer, with manual placements of the laser beam position, the measurement mesh was also designed with an embedded sensor/transducer grid in mind. Given the relatively coarse structure of the mesh proposed, embedded sensor arrays could be implemented relatively easily in common CTWB applications and lead to cost-effective structural health monitoring solutions in places were manual inspection is costly or impractical. In a world marked by resiliency requirements in the context of a changing climate, embedded sensor arrays generating input to modal analysis techniques may be an interesting solution to guarantee the continuity of critical infrastructure.

## Figures and Tables

**Table 1:** Recent work for damage detection with vibration measurements by SDLV. Abbreviations used in this table: MC modal curvature; ANN artificial neural network; SAGA surrogate-assisted genetic algorithm; MS mode shape.

Ref.	Structure	Structure Dimensions [mm]	Fault Size [mm]	Meas. Equipment	Meas. Points	Method
[19]	aluminum plate	1000 × 1000 × 4	cracks of 250 × 2	PSV-400	443 × 443	wavelet and MC
[1]	aluminum plate	400 × 600 × 3	notch of 20 × 20	PSV-400	39 × 39	derivatives MS
[14]	composite plate	150 × 300 × 1	10 × 10	laser scanning	280 × 560	2D wavelet
[15]	aluminum cantilever beam	1000 × 5	50 × 1.3	OFV-353	200	CMS
[16]	GFRP plates	300 × 300	50 × 50	PSV-400	64 × 64	S-transform
[17]	metal plate	150 × 400	circles with diameters of 10, 20, 30, and 40	PSC-400-3	21 × 11	local modal filters and wavelet
[18]	composite plates	250 × 250 × 2.12	70 × 18	PSV-500	26 × 13	ANN, SAGA
[9]	composite laminate plates	550 × 550 × 4	50 × 50 and 100 × 100	SIMULATION	FE: 44 × 44 × 6	2D CWT
[11]	steel plate	560 × 560 × 3.2	80 × 80	two accelerometers 352C33 (PCB)	5 × 5 and FE: 28 × 28	MSD-2D CWT
[12]	composite plates	500 × 250 × 5	25, 75, 125 mm	PCB accelerometer	10 × 5	MC
This work	composite thin-wall beam	major axis:100 mm, minor axis: 60 mm, length = 585 mm	40, 60, 80 mm	Polytec RSV-150	5 × 30	MSD, CMS

**Table 2:** Materials properties used in this work.

Property	Parameters	Value	Unit
Elasticity	E11	32.296	Gpa
modulus	E22 = E33	13.971	GPa
Shear	G12	6.987	GPa
modulus	G13 = G23	5.756	GPa
Poisson’s ratio	v12	0.262	-
	v13 = v23	0.312	-

**Table 3:** Locations of the damage studied in this work.

Fault Type	Shortcut	Location (*p*) [mm]	Location (*z*) [mm]
Longitudinal	*ℓ*40	73	380–420
Longitudinal	*ℓ*60	73	360–420
Longitudinal	*ℓ*80	73	340–420
Transverse	*ℓ*60	180	40–100

**Table 4:** Experimental natural frequencies for the undamaged structure and the different damage cases studied in this work.

	Undamaged	*ℓ*40	*ℓ*60	*ℓ*80	2 Faults
Mode 1 [Hz]	672	672	672	672	669
Mode 2 [Hz]	825	834	831	828	822
Mode 3 [Hz]	885	876	864	855	849
Mode 4 [Hz]	1068	1065	1065	1065	1059
Mode 5 [Hz]	1368	1374	1371	1359	1350
Mode 6 [Hz]	1410	1410	1410	1395	1386

**Table 5:** Comparison of the experimental results reported in the literature for damage detection.

Ref.	Fault Detected	Damage Location Detected	Comments
[19]	yes	yes	Detected length 20–30% smaller than the true length of the fault.
[1]	yes	yes	The detected fault size was approximately correct.
[14]	yes	yes	All faults were detected and located correctly. The fault shape was not detected correctly, and the intensity varies significantly. The size of the faults was approximately correct.
[16]	yes	yes	The fault was detected in individual mode shapes. In the first mode, the signal is weak and noisy signal. In the two higher modes, the detected fault size was approximately correct.
[17]	yes	no	It was not possible to locate or obtain information on the size of the fault.
[18]	yes	yes	Comparing the two methods presented, SAGA presented better results than ANN. Lower accuracy in experimental data compared to numerical study. Mean error reported of the methods was 20% or higher.
[11]	yes	yes	In some experimental signals, there were features that can be mistaken for (false) damage. The size of the fault was overestimated by approximately 150%.
[12]	yes	yes	The size of the fault was approximately correct.
This work	yes	yes	Transverse and longitudinal faults are analyzed simultaneously. The size of the different faults analyzed is correctly detected.

## Abbreviations

The following abbreviations are used in this manuscript:

CSM	Curvature of mode shape
CTWB	Composite thin-wall beam
FID	Fréchet inception distance
LDV	Laser Doppler vibrometer
MSD	Mode shape difference
SHM	Structural health monitoring
SLDV	Scanning laser Doppler vibrometer
